# Detection and potential mechanisms of subclinical left ventricular dysfunction in asymptomatic young adults with Type-2 Diabetes

**DOI:** 10.1186/1532-429X-15-S1-O48

**Published:** 2013-01-30

**Authors:** Jamal N Khan, Emma Wilmot, Melanie J Davies, Trish Gorely, Kamlesh Khunti, Melanie Leggate, Myra Nimmo, Anvesha Singh, Sven Plein, John D Biglands, Gerry P McCann

**Affiliations:** 1 Cardiovascular Sciences, University of Leicester, Leicester, UK; 2 School of Sport Sciences, Loughborough University, Loughborough, UK; 3 Dept of Health Sciences, University of Leicester, Leicester, UK; 4 CMR Clinical Research Group, University of Leeds, Leeds, UK

## Background

There is an epidemic of obesity and Type-2 Diabetes Mellitus (T2DM) in the developed world. Subclinical diastolic dysfunction in diabetes is associated with the development of overt heart failure. Although diabetic cardiomyopathy is well documented in older adults with T2DM there are scarce data on younger adults and no published CMR data. We aimed to assess the prevalence and potential mechanisms of subclinical left ventricular (LV) dysfunction in young T2DM adults compared to healthy lean (LC) and obese controls (OC).

## Methods

40 subjects (20 T2DM [mean T2DM duration 4.7±4.0years], 10 OC, 10 LC) underwent CMR assessment on a Siemens Avanto 1.5T system. LV function and volumes were assessed using SSFP. Circumferential strain was assessed using a high temporal-resolution CSPAMM tagging sequence at 3 slices (basal, mid-cavity, apical). Rest and stress perfusion was assessed quantitatively for perfusion defects and by calculating myocardial perfusion reserve (MPR) using model independent deconvolution at 3 slices. In a subset of the cohort (11 T2DM, 9 OC and 6 LC) T1 mapping with heart-rate correction in a single short-axis slice was performed using a Modified Look-Locker Inversion Recovery (MOLLI) sequence before and 16 minutes post administration of 0.2mmol/kg of Gadovist. Aortic distensibility was assessed on high temporal-resolution gradient echo images.

## Results

**Figure 1:**
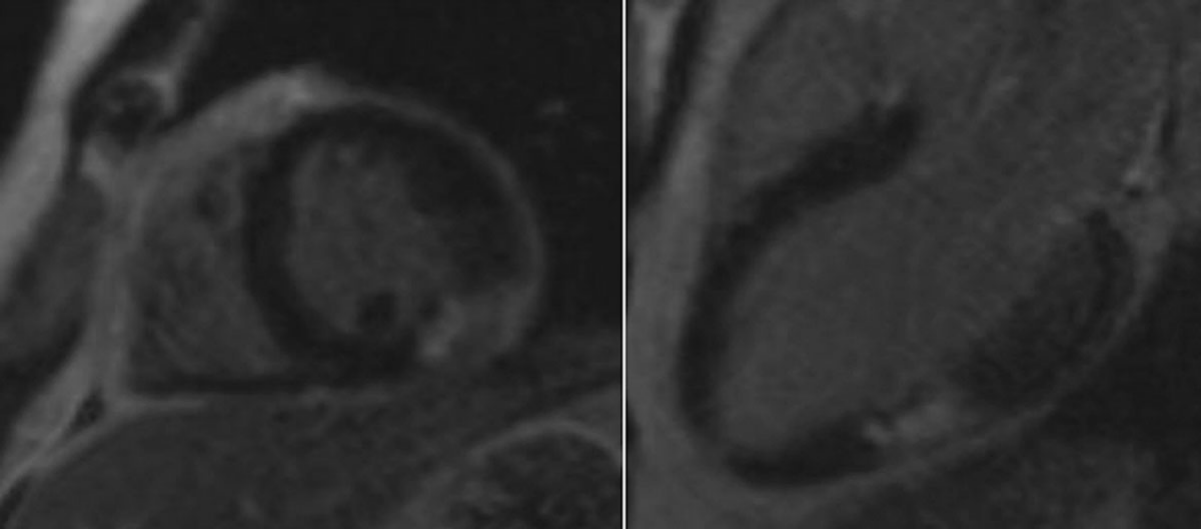
DE imaging short-axis slice (left) and three-chamber long-axis slice (right) in T2DM subject demonstrating significant focal mid-wall enhancement indicative of fibrosis, highlighting the presence of cardiac dysfunction in young diabetics (in this case, the subject had been diabetic for 6 years)

Subjects were well matched for age, height, blood pressure and time of T1 assessment post contrast. Global peak early diastolic strain rate (PEDSR) was significantly lower in T2DM, compared with LC and OC (see table 1). Two patients and 1 OC had evidence of mid-wall late gadolinium enhancement (see figure 1). There was a trend towards concentric remodelling in OC and T2DM associated with significantly reduced PEDSR, particularly in T2DM. There was no regional perfusion defect in any subjects to suggest epicardial coronary artery disease and MPR was similar between the groups. T2DM showed no significant correlation between PEDSR and intracardiac (LV mass, Partition Coefficient of myocardium-blood contrast, MPR), vascular (aortic distensibility), anthropometric (BMI, waist:hip ratio) or metabolic (serum triglycerides, HbA1c, high-sensitivity CRP) markers. However duration of diabetes did inversely relate to PEDSR (r=-0.534, p=0.015).

**Table 1:** CMR and anthropometric data for the 3 study groups (OC, LC, T2DM)

Variable (mean±SD)	Lean control (LC) (n=10)	Obese control (OC) (n=10)	Type-2 diabetes (T2DM) (n=20)	T2DM v LC (p)*	T2DM v OC (p)*
Age (years)	30.0±6.6	30.9±5.6	31.8±6.6	0.871	0.862

Sex (no. M:F)	5:5	6:4	9:11	0.804	0.456

BMI (kg/m2)	21.92±1.71	33.25±2.55	33.86±5.80	<0.001	0.240

BP (mmHg)	131/80±14/12	130/84±15/9	137/88±15/10	0.239	0.255

LVEDM/vol (g/ml)	0.45 (0.42-0.51)	0.54 (0.48-0.60)	0.54 (0.45-0.61)	0.052	0.816

LVEF (%)	55.50±3.53	53.64±4.46	54.85±5.05	0.727	0.481

PEDSR (1/s) (n for analysis)	1.80±0.47 (n=9)	1.59±0.32 (n=9)	1.27±0.34 (n=16)	0.006	0.026

Global MPR	3.68±1.57	3.03±0.75	3.57±1.08	0.927	0.270

Partition Coefficient (n for analysis)	0.443±0.05 (n=6)	0.442±0.06 (n=9)	0.443±0.06 (n=11)	0.112	0.778

## Conclusions

This study does not suggest that obesity, ischaemia, microvascular dysfunction or diffuse myocardial fibrosis are the main mechanisms contributing to subclinical diastolic dysfunction in young adults with T2DM. Duration of T2DM was strongly associated with diastolic dysfunction. Young onset T2DM is likely to result in a very high lifetime risk of cardiovascular events and heart failure.

## Funding

NIHR Leicester-Loughborough Diet, Lifestyle and Physical Activity BRU at the University Hospitals of Leicester NHS Trust, University of Leicester and Loughborough University, the NIHR Leicester Cardiovascular Biomedical Research Unit and the NIHR Collaboration for Leadership in Applied Health Research and Care - Leicestershire, Northamptonshire and Rutland (NIHR CLAHRC for LNR).

